# Mycoepoxydiene Inhibits Lipopolysaccharide-Induced Inflammatory Responses through the of TRAF6 Polyubiquitination

**DOI:** 10.1371/journal.pone.0044890

**Published:** 2012-09-11

**Authors:** Qiang Chen, Tenghui Chen, Wenjiao Li, Wei Zhang, Jingwei Zhu, Yang Li, Yaojian Huang, Yuemao Shen, Chundong Yu

**Affiliations:** 1 State Key Laboratory of Cellular Stress Biology, School of Life Sciences, Xiamen University, Xiamen, Fujian, China; 2 School of Pharmaceutical Sciences, Shandong University, Jinan, Shandong, China; Ohio State University, United States of America

## Abstract

Mycoepoxydiene (MED) is a polyketide isolated from a marine fungus associated with mangrove forests. MED has been shown to be able to induce cell cycle arrest and cancer cell apoptosis. However, its effects on inflammatory response are unclear. Herein we showed that MED exhibited inhibitory effect on inflammatory response induced by lipopolysaccharide (LPS). MED significantly inhibited LPS-induced expression of pro-inflammatory mediators such as tumor necrosis factor-α (TNF-α), interleukin (IL)-1β, IL-6, and nitric oxide (NO) in macrophages. MED inhibited LPS-induced nuclear translocation of nuclear factor (NF)-κB (NF-κB) p65, IκB degradation, IκB kinase (IKK) phosphorylation, and the activation of extracellular signal-regulated kinase (ERK), c-jun N-terminal kinase (JNK), and p38, suggesting that MED blocks the activation of both NF-κB and mitogen-activated protein kinase (MAPK) pathways. Furthermore, the effects of MED on LPS-induced activation of upstream signaling molecules such as transforming growth factor-β–activated kinase 1 (TAK1), tumor necrosis factor receptor-associated factor 6 (TRAF6) and IL-1 receptor associated kinases1 (IRAK1) were investigated. MED significantly inhibited TAK1 phosphorylation and TRAF6 polyubiquitination, but not IRAK1 phosphorylation and TRAF6 dimerization, indicating that MED inhibits LPS-induced inflammatory responses at least in part through suppression of TRAF6 polyubiquitination. Moreover, MED protected mice from LPS-induced endotoxin shock by reducing serum inflammatory cytokines. These results suggest that MED is a potential lead compound for the development of a novel nonsteroidal anti-inflammatory drug.

## Introduction

Lipopolysaccharide (LPS), an essential component of the outer membrane of Gram-negative bacteria, can trigger host inflammatory responses which are critical for host defense against bacterial invasion [1], [2]. However, overwhelming and uncontrolled inflammatory responses can lead to severe endotoxin shock and even death [2]. Toll-like receptors (TLRs) recognize a variety of pathogen-associated molecular patterns to initiate various signaling pathways leading to inflammation [3]. LPS induces inflammatory responses via binding to TLR4 and triggering cascades of intracellular signaling events to promote the secretion of pro-inflammatory mediators including tumor necrosis factor-α (TNF-α), interleukin (IL)-1β, IL-6, and nitric oxide (NO) [1], [4]. These pro-inflammatory mediators play an important role in the endotoxin shock and organ failure during inflammatory responses [5].

It has been well demonstrated that nuclear factor-κB (NF-κB) and mitogen-activated protein kinases (MAPKs) including extracellular signal-regulated kinase (ERK), c-jun N-terminal kinase (JNK), and p38 are major signaling pathways that mediate LPS-induced inflammatory responses [6]. LPS-elicited activation of NF-κB and MAPK pathways is initiated by the interaction between myeloid differentiation factor 88 (MyD88) and Toll-like receptor 4 (TLR4) upon LPS engagement. Then, MyD88 recruits IL-1 receptor-associated kinases (IRAKs), tumor necrosis factor receptor-associated factor 6 (TRAF6), and the transforming growth factor-β-activated kinase 1 (TAK1) complex, leading to early-phase activation of NF-κB and MAPKs [7]. NF-κB, an important transcriptional factor, normally locates in the cytoplasm forming an inactive complex with its inhibitory protein IκBα. Upon stimulation, IκB kinase (IKK), which is activated by TAK1 complex, can phosphorylate IκBα. Subsequently, phosphorylated IκBα is degraded by the proteasome to allow translocation of NF-κB into the nucleus to initiate specific target gene transcription [8]. MAPKs, which are activated within the protein kinase cascades consisting of three enzymes MAP kinase, MAP kinase kinase (MAP2K) and MAP kinase kinase kinase (MAP3K) [9], can activate downstream transcription factors such as c-Jun, c-Fos, ATF2, and Elk-1 to promote the expression of pro-inflammatory mediators. Thus, blockage of the abovementioned signaling pathways by small molecules represents a promising therapeutic strategy for inflammation-associated diseases [10]–[13]. For examples, aspirin and salicylate target IKKβ to suppress the initiation and propagation of inflammation [14], specific inhibitors for ERK, JNK and p38 pathways reverse inflammatory responses [11]–[13], and TH-4-PX targets IRAK-1 to execute anti-inflammatory action [15].

Mycoepoxydiene (MED), a novel polyketide containing an oxygen-bridged cyclooctadiene core and an α, β-unsaturated δ lactone moiety [16], was isolated from the marine fungus *Diaporthe* sp. (*D.* sp) HLY-1 found in submerged rotten leaves of Kandelia candel in a mangrove forest in Fujian Province, China [17]. A similar natural compound, whose biological function still has not been described, was isolated from the fermentation broth of OS-F66617, a fungal strain obtained from the deadwood of forest in Brazil [18]. Previous studies showed that MED has antimicrobial and anticancer activities [17], [19]. In human cervical cancer cell (Hela cell), MED maintains an anti-growth characteristic through accelerating cytoskeletal rearrangement, inducing cell cycle arrest in G2/M phase, prompting the activities of p38 and JNK signaling pathways, and increasing the release of cytochrome C as well as caspase-3 mediated cell apoptosis [19]. Despite some biological significances of MED have been revealed, its effects on inflammatory response are unclear. In this study, we demonstrated that MED inhibited LPS-induced inflammatory responses through blocking the activation of NF-κB and MAPK pathways via the suppression of TRAF6 polyubiquitination. Additionally, MED effectively prevented LPS-induced endotoxin shock in mice.

## Results

### MED strongly inhibits LPS-induced inflammatory response in macrophages

**Figure 1:**
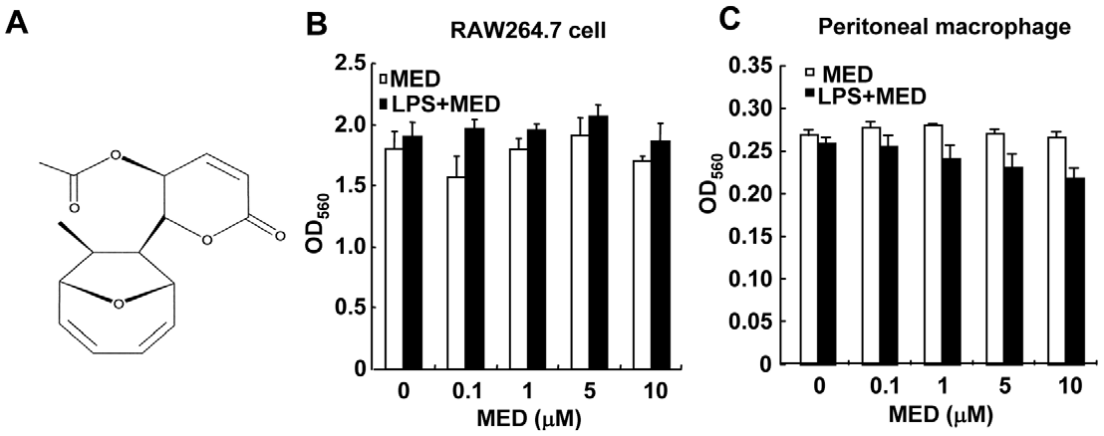
Effect of MED on the viability of macrophages. A. Chemical structure of MED. B, C. RAW264.7 cells (B) and primary peritoneal macrophages (C) were treated with various doses of MED in the presence or absence of LPS (100 ng/ml) for 24 h. Cell viability was determined as described in the Methods. Data shown are the mean + SD (n = 3).

MED is a novel polyketide containing an oxygen-bridged cyclooctadiene core and an α, β-unsaturated δ-lactone moiety (Figure 1A). MED has been shown to be able to induce tumor cell apoptosis [19]. However, MED had no apparent cytotoxicity to murine macrophage cell lines RAW264.7 and primary peritoneal macrophages at concentrations up to 10 μM in the absence or presence of LPS (p>0.05, Figure 1B and C).

**Figure 2:**
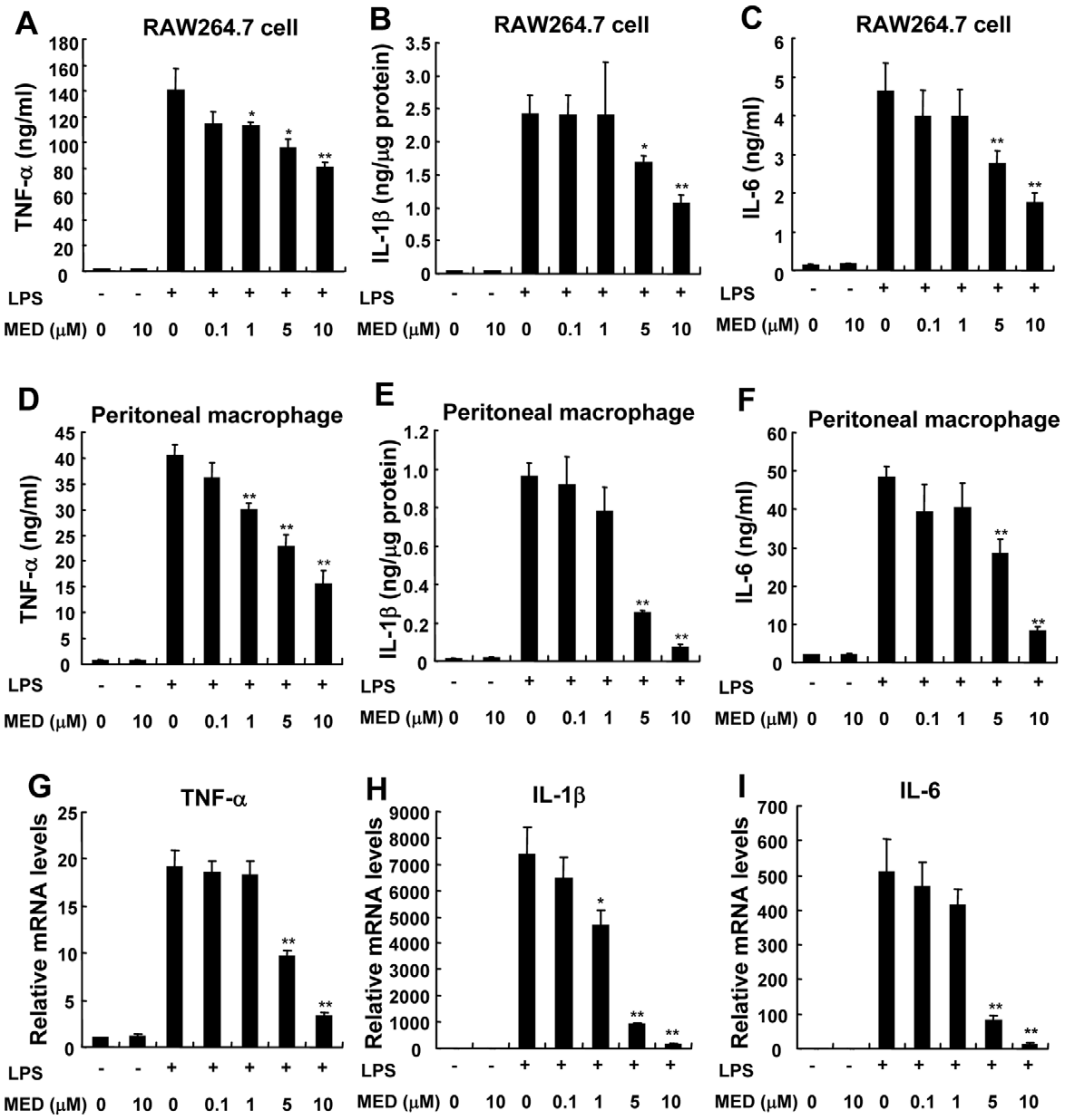
MED inhibits the production of pro-inflammatory cytokines in RAW264.7 cells and primary peritoneal macrophages. A–F. RAW264.7 cells (A–C) or peritoneal macrophages (D–F) were treated with LPS (100 ng/ml) together with various doses of MED or MED alone for 6 h. The levels of TNF-α (A, D), IL-1β (B, E), and IL-6 (C, F) were determined using ELISA kits. Data shown are the mean + SD (n = 3). *p*<*0.05, **p*<*0.01. G-I. RAW264.7 cells were treated with LPS (100 ng/ml) together with various doses of MED or MED alone for 4 h. The mRNA levels of TNF-α (G), IL-1β (H), and IL-6 (I) were measured by real-time PCR. Data shown are the mean + SD (n = 3). * p*<*0.05, ** p*<*0.01.

To examine whether MED can affect inflammatory response induced by LPS, pro-inflammatory cytokines such as TNF-α, IL-1β, and IL-6 were measured after LPS stimulation in the presence or absence of MED. As shown in Figure 2A–F, the levels of TNF-α, IL-1β, and IL-6 induced by LPS were significantly inhibited by MED in a dose-dependent manner in RAW264.7 cells as well as in primary peritoneal macrophages. Furthermore, MED could significantly suppress the mRNA levels of TNF-α, IL-1β and IL-6 induced by LPS (Figure 2G–I), indicating that MED inhibits LPS-induced cytokine expression at the transcriptional level.

**Figure 3:**
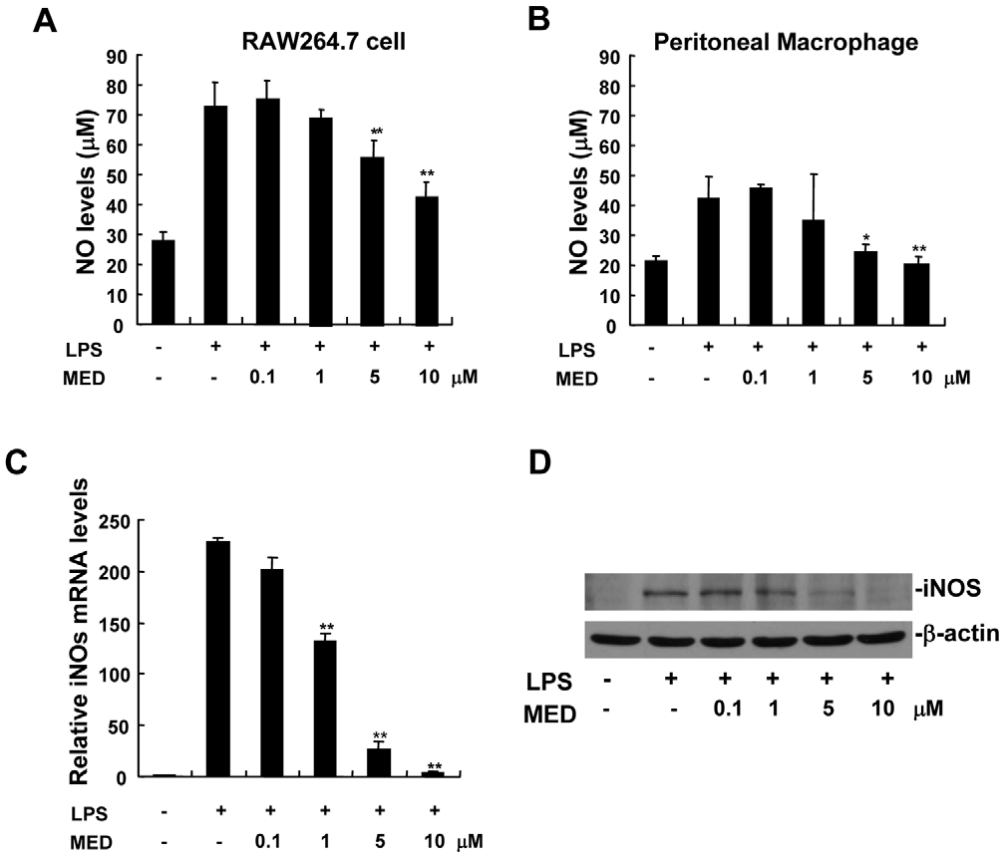
MED inhibits NO production and iNOS expression in RAW264.7 cells as well as in primary peritoneal macrophages. A, B. RAW264.7 cells (A) or peritoneal macrophages (B) were treated with LPS (100 ng/ml) together with various doses of MED for 24 h. The level of NO was determined using commercially available kit. Data shown are the mean + SD (n = 3). * p*<*0.05, ** p*<*0.01. C. RAW264.7 cells were treated with LPS (100 ng/ml) together with various doses of MED for 6 h, and the mRNA levels of iNOS were measured by real-time PCR. Data shown are the mean + SD (n = 3). ** p*<*0.01. D. RAW264.7 cells were treated with LPS (100 ng/ml) together with various doses of MED for 24 h, and the protein levels of iNOS were determined by Western blotting.

Nitric oxide (NO) is known to function as a pro-inflammatory mediator in the pathogenesis of inflammation [20]. NO is synthesized by nitric oxide synthases (NOS) including three isozymes such as neuronal NOS (nNOS), endothelial NOS (eNOS) and inducible NOS (iNOS). nNOS and eNOS are constitutive NOS enzymes, iNOS is inducible and involved in immune response by generating NO [21]. As shown in Figure 3A and B, NO was induced in RAW264.7 cells as well as in primary peritoneal macrophages after LPS treatment, but MED significantly inhibited LPS-induced NO production at the concentrations of 5 and 10 μM. Furthermore, the effect of MED on iNOS expression induced by LPS was measured by real-time PCR and Western blotting. As shown in Figure 3C, MED dose-dependently inhibited LPS-induced expression of iNOS mRNA. Consistently, MED suppressed LPS-stimulated iNOS protein production in a dose-dependent manner (Figure 3D).

### MED effectively suppresses LPS-induced NF-κB activation

**Figure 4:**
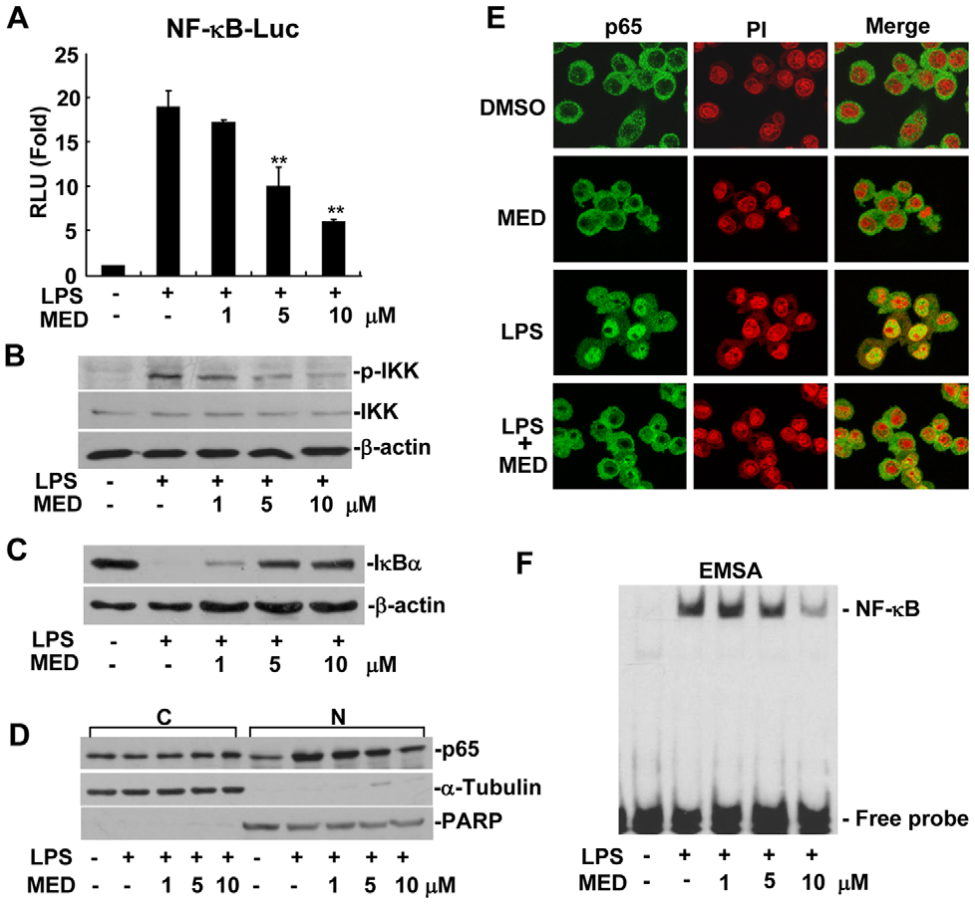
MED suppresses LPS-induced NF-κB activation in RAW264.7 cells. A. Effect of MED on NF-κB luciferase reporter activity after LPS treatment. Cells were transiently transfected with NF-κB luciferase reporter plasmids and incubated overnight, then cells were treated with LPS (100 ng/ml) together with various doses of MED for 6 h. Luciferase activities were determined as described in the Methods. Data shown are the mean + SD (n = 3). **p*<*0.01. B. Effect of MED on phosphorylation of IKK induced by LPS. Cells were treated with LPS (100 ng/ml) together with various doses of MED for 10 min. C. Effect of MED on degradation of IκBα induced by LPS. Cells were treated by LPS (100 ng/ml) together with various doses of MED for 15 min. D, E. Effect of MED on nuclear translocation of NF-κB p65 induced by LPS. Cells were treated by LPS (100 ng/ml) together with various doses of MED for 30 min, then nuclear translocation of p65 was determined by Western blotting (D) or Immunofluoresence analysis (E). C: Cytoplasm; N: Nucleus. The area of nucleus was marked with PI. F. Effect of MED on DNA binding activity of NF-κB induced by LPS. Cells were treated with LPS (100 ng/ml) together with various doses of MED for 30 min. Nuclear extracts were obtained for EMSA.

NF-κB pathway plays an important role in regulating the expression of pro-inflammatory factors such as TNF-α, IL-1β, IL-6, and iNOS during inflammatory response. To investigate whether the inhibition of pro-inflammatory factors by MED is attributed to the suppression of this pathway, NF-κB dependent promoter activity was examined in the presence or absence of MED by using a luciferase reporter assay. As shown in Figure 4A, MED significantly inhibited NF-κB luciferase reporter activity induced by LPS in RAW 264.7 cells at the concentrations of 5 and 10 μM, suggesting that MED inhibits LPS-induced NF-κB activation.

A series of upstream regulatory signaling factors such as IRAK1, TRAF6, TAK1, and IKK mediate LPS-induced NF-κB activation. Activated IKK can directly phosphorylate IκB, and then lead to its degradation to allow translocation of NF-κB into the nucleus to initiate target gene transcription [8]. Therefore, we examined whether MED can suppress IKK activation and prevent IκBα degradation by modulating LPS-induced IKK phosphorylation. As shown in Figure 4B and C, MED inhibited LPS-induced IKK phosphorylation and IκBα degradation in a dose-dependent manner. Furthermore, we investigated the effect of MED on the nuclear translocation of NF-κB. In the absence of MED, LPS induced a significant nuclear translocation of p65 subunit of NF-κB, but the amount of p65 translocated into the nucleus was significantly reduced in the presence of MED (Figure 4D and E). In addition, MED decreased LPS-induced DNA binding activity of NF-κB at the concentrations of 5 and 10 μM (Figure 4F and Figure S1). These results demonstrate that MED suppresses LPS-induced NF-κB activation through inhibiting the activation of IKK.

### MED effectively suppresses LPS-induced activation of MAPK pathways

**Figure 5:**
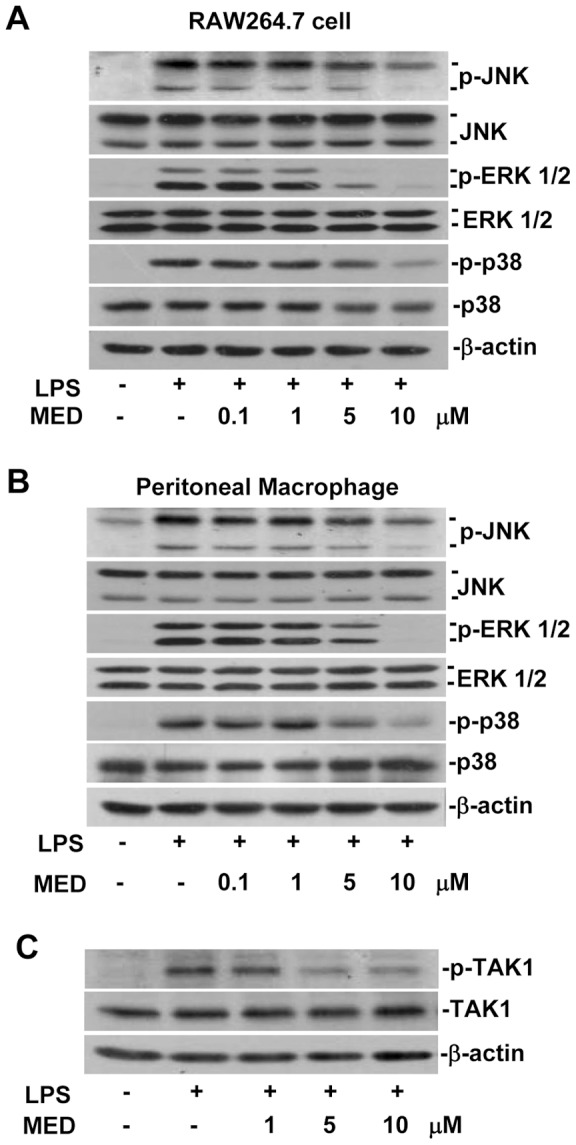
MED suppresses LPS-induced MAPK activation. A, B. RAW264.7 cells (A) and primary peritoneal macrophages (B) were treated with LPS (100 ng/ml) together with various doses of MED for 20 min. The phosphorylation of MAPKs including JNK, ERK1/2 and p38 was determined by Western blotting analysis. C. Effect of MED on phosphorylation of TAK1 induced by LPS in RAW264.7 cells. Cells were treated with LPS (100 ng/ml) together with various doses of MED for 10 min.

In addition to NF-κB pathway, MAPK pathways which include ERK, p38, and JNK are also involved in LPS-induced production of inflammatory mediators in macrophages [6]. Therefore, the effect of MED on the activation of MAPK pathways was examined by detecting the phosphorylation of these kinases. As shown in Figure 5A and B, MED significantly suppressed LPS-induced phosphorylation of JNK, ERK1/2, and p38 in RAW264.7 cells as well as in primary peritoneal macrophages at the concentrations of 5 and 10 μM, indicating that MED can suppress LPS-induced activation of MAPK pathways.

Since TAK1 has been shown to be a pivotal factor for the activation of IKK, JNK, and p38 in response to TLR stimulation [22], and MED can suppress LPS-induced activation of IKK, JNK, and p38, the effect of MED on the activation of TAK1 was examined. As shown in Figure 5C, MED significantly inhibited LPS-induced TAK1 activation at the concentrations of 5 and 10 μM, demonstrating that MED effectively suppresses the activation of IKK, JNK and p38 through inhibiting the activation of TAK1.

### MED effectively suppresses LPS-induced polyubiquitination of TRAF6

**Figure 6:**
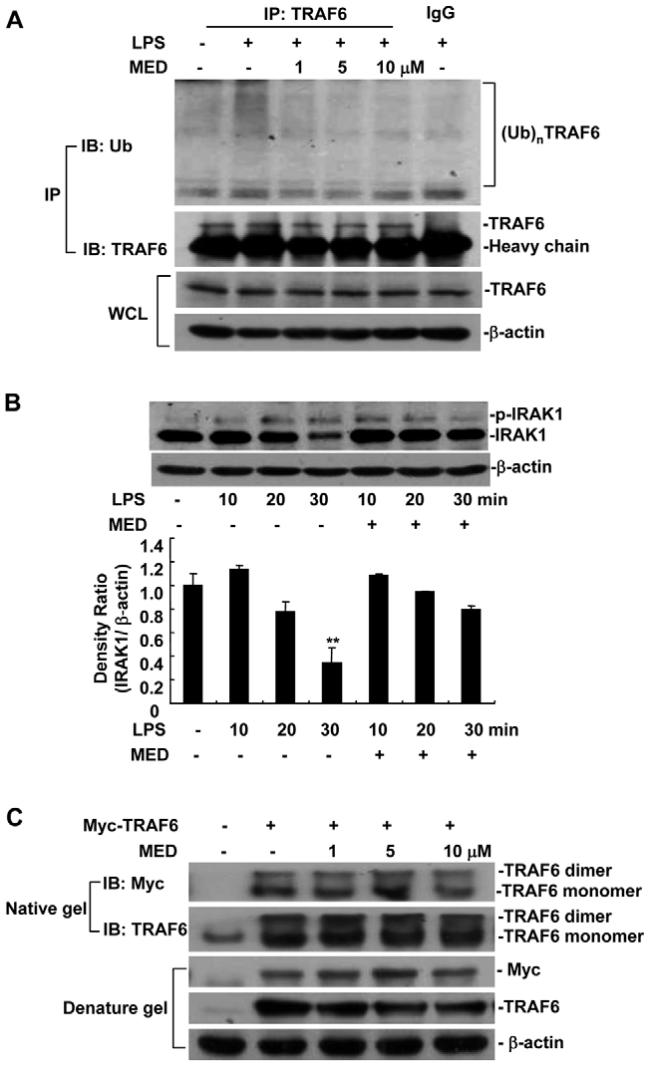
MED suppresses TRAF6 polyubiquitination but not IRAK1 phosphorylation and TRAF6 dimerization. A. Effect of MED on polyubiquitination of TRAF6 induced by LPS in RAW264.7 cells. Cells were treated with LPS (100 ng/ml) together with various amounts of MED for 10 min. The cell lysates were immunoprecipitated by TRAF6 antibodies or isotype IgG, and then TRAF6 polyubiquitination was determined by Western blotting. B. Effect of MED on activation of IRAK1 induced by LPS in RAW264.7 cells. Cells were treated by LPS (100 ng/ml) together with MED (10 μM) for different times. The non-phosphorylated and phosphorylated IRAK1s (active form) were detected by Western blotting. ** p*<*0.01. C. Effect of MED on dimerization of TRAF6 in HEK 293T cells overexpressing TRAF6. The cells were transfected with Myc-TRAF6 expression plasmids and incubated overnight, then the cells were treated with various doses of MED for 4 h. Cell lysates were analyzed by native gel and denature gel.

It has been reported that TRAF6 acts as an upstream regulator of TAK1 in TLR4 signaling pathway. LPS induces phosphorylation of IRAK1 and then promotes interaction between IRAK1 and TRAF6, resulting in TRAF6 dimerization, which leads to Lys 63 polyubiquitination of target proteins including TRAF6 itself. Ubiquitinated TRAF6 recruits TAB2 and activates the TAB2-associated TAK1 kinase, which then phosphorylates and activates IKK [23], [24]. TRAF6 deficiency results in defective activation of NF-κB and MAPK pathways [25], suggesting that TRAF6 is essential for LPS-induced inflammatory response. Our result also showed that TRAF6 knockdown could inhibit activation of NF-κB and MAPK induced by LPS (Figure S2). Therefore, we investigated whether MED suppresses LPS-induced polyubiquitination of TRAF6. The results showed that MED markedly inhibited LPS-induced TRAF6 polyubiquitination (Figure 6A). Furthermore, we examined the activation of IRAK1, which is required for TRAF6 polyubiquitination, in response to LPS stimulation. IRAK1 is activated by phosphorylation and then undergoes proteasome-mediated degradation after LPS stimulation [26]. Phosphorylation of IRAK1 leads to a slower migrating band of IRAK1 in SDS-PAGE [26], [27]. As shown in Figure 6B, MED had no significant effects on the phosphorylation of IRAK1 at 10, 20, and 30 min after LPS treatment, although MED markedly inhibited the degradation of IRAK1 at 30 min after LPS treatment. In addition, we investigated whether MED could block dimerization of TRAF6 in HEK 293T cells overexpressing TRAF6. The result showed that MED did not affect TRAF6 dimerization (Figure 6C). Collectively, these results suggest that MED inhibits LPS-induced inflammatory response through blocking the polyubiquitination of TRAF6 independently on the activation of IRAK1.

**Figure 7:**
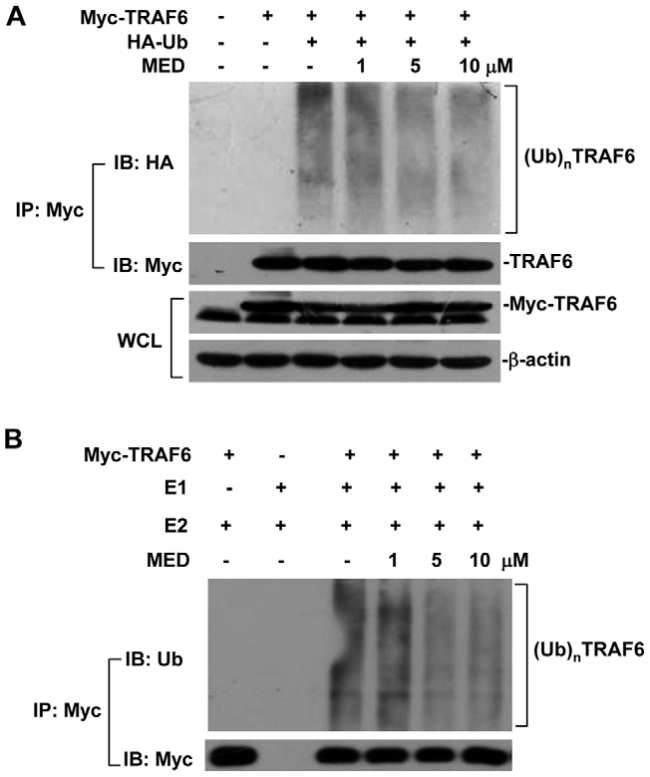
Effect of MED on the ubiquitination of TRAF6 *in vivo* and *in vitro*. A. HEK 293T cells were transfected with Myc-TRAF6 and HA-Ub expression plasmids and incubated overnight, then cells were treated with various doses of MED for 4 h. Cell lysates were immunoprecipitated and analyzed by Western blotting. B. MED inhibited the ubiquitination of TRAF6 *in vitro* ubiquitination assay. HEK 293T cells were transfected with Myc-TRAF6 expression plasmids and incubated overnight, and then cell lysates were immunoprecipitated by TRAF6 antibodies for 3 h. Then the beads were washed three times using IP buffer and twice using TBS buffer. The beads were pre-treated with various doses of MED for 30 min on ice and then subjected to *in vitro* ubiquitination assay.

To determine whether MED can inhibit the ubiquitination of TRAF6 independently on TLR4 signaling, we performed the ubiquitination assays in 293T cells without LPS treatment. As shown in Figure 7A, MED blocked the ubiquitination of TRAF6 in a dose-dependent manner. Furthermore, MED also markedly suppressed the ubiquitination of TRAF6 *in vitro* ubiquitination system (Figure 7B). These results indicate that MED may be a direct ubiquitination inhibitor for TRAF6 unbiquitination process.

### MED effectively protects mice from LPS-induced endotoxin shock

**Figure 8:**
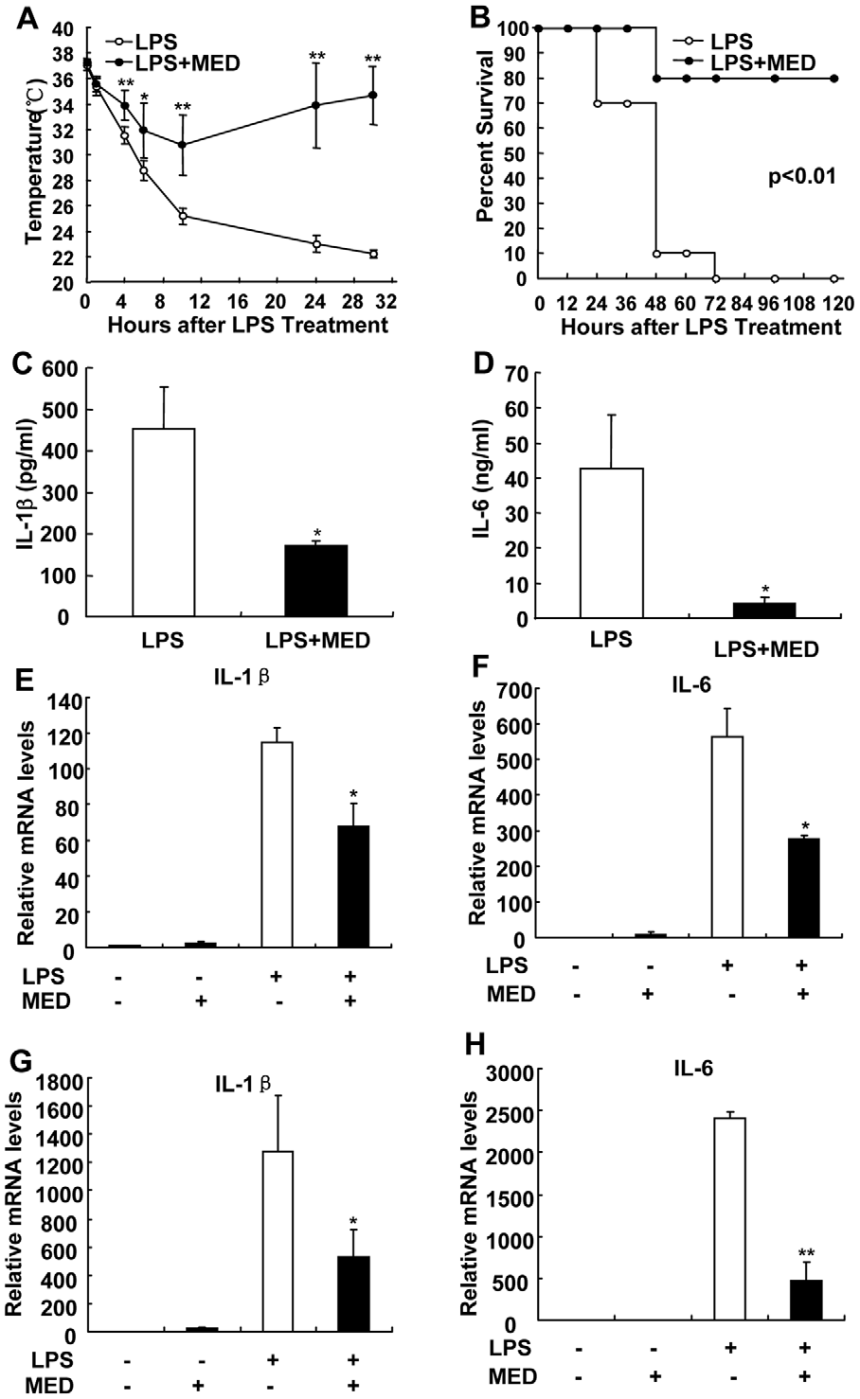
MED protects mice from LPS-induced endotoxin shock. A. BALB/c mice were divided in two groups (n = 10 for each group), one group was only i.p. injected with LPS (10 mg/kg); another group was pre-injected with MED (15 mg/kg) for 30 min before LPS injection. Body temperatures of two groups of mice were measured at different time points. B. Survival rates of mice were examined after LPS administration with or without MED pretreatment. C, D. The levels of IL-1β (C) and IL-6 (D) in serum were measured at 8 h after LPS injection with or without MED pretreatment. *p*<*0.05, **p*<*0.01. E, F. MED reduced the mRNA levels of IL-1β and IL-6 in the liver of mice after LPS treatment. G, H. MED reduced the mRNA levels of IL-1β and IL-6 in peritoneal macrophages isolated from mice treated with LPS.

The observations that MED effectively inhibited LPS-induced inflammatory responses *in vitro* prompted us to investigate whether MED can inhibit LPS-induced endotoxin shock *in vivo*. In the absence of LPS, MED treatment did not affect the body temperatures and serum cytokine levels in mice (Figure S3). However, after 10 mg/kg LPS administration, the body temperature of control mice markedly decreased and dropped to 22 °C at 30 h, indicating that mice suffer from severe LPS-induced endotoxin shock. In contrast, pretreatment of MED effectively suppressed the drop of body temperature caused by LPS (Figure 8A). All mice administrated with LPS alone died within 72 h, but 80% of mice pretreated with MED survived and recovered by the end of experiment (Figure 8B). Consistently, MED significantly suppressed the levels of serum IL-1β and IL-6 (Figure 8C, D).

Furthermore, we detected the mRNA levels of IL-1β and IL-6 in the livers which contain many Kupffer cells (liver macrophages) as well as in isolated peritoneal macrophages after treatment with LPS with or without MED. As shown in Figure 8E–H, MED inhibited the expression of these cytokines in the livers and in isolated peritoneal macrophages after treatment with LPS, confirming the inhibitory effects of MED on LPS-induced inflammation is the result of inhibition of macrophage cytokine expression *in vivo*.

## Discussion

MED, a novel polyketide, was isolated from the marine fungus *D.* sp. HLY-1 found in submerged rotten leaves of *Kandelia candel* in a mangrove forest [17]. Previous studies showed that MED has antimicrobial and anticancer activities [17], [19]. In the present study, we showed that MED not only markedly inhibited LPS-induced inflammatory response in cultured macrophages, but also suppressed septic shock induced by LPS *in vivo*, demonstrating that MED has an anti-inflammatory activity.

Stimulation of TLR4 by LPS activates intracellular signaling events such as IKK/NF-κB and MAPK pathways to induce the production of various inflammatory mediators [6]. MAPKs belong to a highly conserved family of protein serine/threonine kinases including p38, JNK and ERK1/2, whereas IKK is a kinase for NF-κB activation. Suppression of the activation of p38, JNK, ERK1/2, and IKK by MED suggests that MED could affect upstream signaling of IKK and MAPKs. TAK1, a member of the MAP3K family, is known to act as a key modulator of IKK pathway as well as p38 and JNK cascades [22]. MED suppressed the activation of TAK1 induced by LPS, indicating that MED negatively affects IKK, p38, and JNK pathways through blocking the activation of TAK1.

Binding of LPS to TLR4 results in recruitment of MyD88 to the membrane, and then promotes the assembly of a proximal signaling complex that includes IRAK1, IRAK4 and TRAF6, which facilitates the phosphorylation of IRAK1 by IRAK4 [28], [29]. Phosphorylated activation of IRAK1 promotes dissociation of IRAK1 and TRAF6 from TLR4. Activated IRAK1 is ubiquitinated and eventually degraded by the proteasome, which serves as a negative feedback mechanism of down-regulating IL-1R/TLR-mediated signaling and cytokine gene transcription [30]. The dimerization of TRAF6 catalyzes the formation of a polyubiquitin chain linked through Lys63, which is required for activation of TAK1 in LPS-induced cellular responses [23]. MED suppressed the polyubiquitination of TRAF6, but did not affect TRAF6 dimerization and IRAK1 phosphorylation, suggesting that TRAF6 could be the target of MED in response to LPS. Ubiquitination plays an important role in regulation of TLR signaling pathway including positive and negative feedback [23]. MED inhibited not only polyubiquitination of TRAF6 but also polyubiquitination-mediated degradation of IRAK1 and IκBα induced by LPS, implicating that MED may serve as an ubiquitination inhibitor to inhibit the ubiquitination of proteins involved in TLR4 pathway. Indeed, we found that MED significantly blocked the ubiquitination of TRAF6 and IκBα (Figure 7A and Figure S4) in HEK 293T cells without LPS treatment, indicating that MED can inhibit protein ubiquitination independently on TLR4 signaling. The mechanism by which MED inhibits protein ubiquitination is currently under investigation.

**Figure 9:**
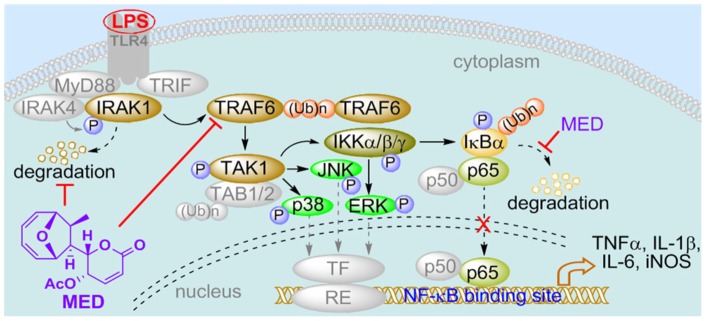
Schematic representation of the mechanism by which MED inhibits LPS-induced inflammatory responses in macrophages. MED blocks LPS-induced polyubiquitination of TRAF6 and then inhibits the activation of TAK1 and IKK, leading to the inhibition of multi-signaling cascades including NF-κB and MAPKs such as p38, JNK and ERK, that in turn leads to inhibition of cytokine/iNOS induction. In addition, MED may be involved in regulating the ubiquitination and degradation of other proteins such as IRAK1 and IκBα to affect LPS signaling.

Collectively, in this study, we demonstrate that MED, as a novel marine microbial compound, can not only effectively inhibit LPS-induced inflammatory response through the suppression of TRAF6 polyubiquitination (Figure 9), but also alleviate LPS-induced endotoxin shock *in vivo*, suggesting that MED is a potential lead compound for the development of novel anti-inflammatory drugs, and marine microbial bioactive compounds may be an important source of new anti-inflammatory drugs.

## Materials and Methods

### Materials

LPS (*Escherichia coli* strain O111:B4) was obtained from Sigma (St Louis, MO, USA). MED was isolated from the fermentation broth of *D.* sp. HLY-1 as described [17]. The identity of MED was confirmed by HRMS, ^1^H and ^13^C NMR analysis, as reported by Lin *et* *al*. [17]. The purity of MED exceeded 95.7% according to the HPLC analysis (Figure S5). A 10 mM stock solution in DMSO was prepared and stored at −20 °C. Antibodies against phospho-p38, p38, phospho-JNK, JNK, phospho-ERK 1/2, ERK 1/2, phospho-IKKα/β, IKKβ, phospho-Akt, Akt, phospho-TAK1 and IRAK1 were purchased from Cell Signaling Technology (Danvers, MA, USA). Antibodies against IκBα, p65, inducible NO synthase (iNOS), PARP, α-tubulin, TAK1, TRAF6 and ubiquitin (Ub) were obtained from Santa Cruz Biotechnology (Santa Cruz, CA, USA). β-actin antibody was purchased from Sigma. Female BALB/c mice (6–8 weeks age) were used for all experiments. Animals were maintained with specific pathogen free air at a temperature between 20 and 23 °C with 12 h light and dark cycles and relative humidity of 50%. Animal experiments were performed in accordance with the Guide for the Care and Use of Laboratory Animals. All animal experimental procedures were approved by Animal Care and Use Committee of Xiamen University (Protocol Number: XMULAC20120001). Every effort was made to reduce the suffering of animals.

### Cell culture and transfection

Murine macrophage cell line RAW264.7 cells and human embryonic kidney cell line HEK293T were purchased from the American Type Cell Culture Collection. Peritoneal macrophages were obtained from mice after three days' 4% Thioglycollate medium (Sigma) induction. All cell lines were cultured in DMEM supplemented with 10% fetal bovine serum (FBS). Cells were transfected with different plasmids using lipofectamine 2000 (Invitrogen, Carlsbad, CA) and with siRNA (100 nM) using *Trans*IT-TKO transfection reagent (Mirus, Madison, WI) according to the manufacturer's instructions. The TRAF6-specific siRNA were 5′-ACCACGAAGAGGUCAUGGA-3′ (sense) and 5′-UCCAUGACCUCUUCGUGGU-3′ (antisense).

### Cell viability assay

Cell viability was analyzed by MTT assay. A total of 8×10^4^ cells were seeded in 96 well plates. After incubation of 24 h, 10 µl MTT (5 mg/ml, Sigma) was added to each well. The plates were incubated for 4 h before addition of 100 µl lysis buffer (10% SDS in 0.01 M HCl). The absorbance was measured at 560 nm using a microplate reader.

### Cytokine assay

Cells were seeded in 96 well plates 24 h prior to LPS and MED treatment. After added with LPS or LPS plus MED, cells were further cultured for 6 h. The concentrations of TNF-α, IL-1β, and IL-6 were determined using ELISA kits (eBioscience, San Diego, CA) according to the manufacturer's instructions.

### Nitrite measurement

Cells were seeded in 96 well plates 24 h prior to LPS and MED stimulation. After addition LPS or LPS plus MED, cells were further cultured for 24 h. The concentration of NO was determined by commercially available kit (Nanjing Jiancheng Bioengineering Institute, Nanjing, China) according to the manufacturer's instruction.

### Luciferase reporter assay

Cells were harvested 24 h after transfection and luciferase activity was assayed and normalized to Renilla luciferase activity using a dual luciferase reporter assay system (Promega, Madison, WI).

### Nuclear protein extraction

After LPS and MED treatments, cells were collected and added with Buffer A (10 mM Hepes (pH 7.9), 10 mM KCl, 0.1 mM EDTA, 1 mM DTT, and 0.5 mM PMSF). After incubated in ices for 20 min, cells were added with Buffer A with 2.5% NP- 40 and vibrated for 10 sec and then centrifuged at 12000 g for 5 min at 4 °C, the supernatants were collected to detect proteins located in cytoplasm. Buffer B(20 mM Hepes (pH 7.9), 0.4 M NaCl, 1 mM EDTA, 1 mM DTT, 1 mM PMSF)was added to the precipitates and vibrated for 25 min at 4 °C and then centrifuged at 12000 g for 5 min at 4 °C, the supernatants were collected as nuclear extracts, and the protein concentration was measured by Bradford protein assay (Bio-Rad, Hercules, CA).

### Immunofluoresence for NF-κB p65 localization

Cells were treated with LPS and MED for 30 min and then fixed with 4% paraformaldehyde for 15 min. After penetrated with 0.3% Triton X-100 in PBS for 10 min, cells were blocked with 5% bovine serum albumin for 30 min and incubated with Rabbit polyclonal anti-p65 antibodies for 2 h. Then, cells were incubated with Fluorescein-conjugated Goat anti-Rabbit IgG for 1 h and counterstained with propidium iodide (PI) for 5 min. Stained slides were mounted with mounting medium and analyzed under a laser confocal fluorescence microscopy (Leica, Heidelberg, Germany).

### Electrophoretic mobility shift assay

Five µg of nuclear extract was incubated with 20 nM biotin-labeled double strands oligonucleotide probes for 20 min at room temperature and then separated on a non-denaturing 6% (w/v) polyacrylamide gel. The biotin-labeled oligonucleotide probes were transferred from the polyacrylamide gel onto a nylon membrane and then detected with LightShift chemiluminescent EMSA kit (Pierce, Rockford, IL) according to the manufacturer's instruction.

### Quantitative real-time PCR

Total RNA was isolated with Trizol reagent (Invitrogen) according to the manufacturer's instructions. The cDNA was synthesized from 2 μg of total RNA using MMLV transcriptase (ToYoBo, Shanghai, China) with random hexamers. Real-time PCRs were performed using SYBR Premix ExTaq (TaKaRa, Dalian, China). Relative quantification was achieved by normalization to the amount of TBP. Primers used for real-time PCR are listed on Table 1.

**Table 1:** Primers for real-time PCR.

Gene	Forward Primer	Reverse Primers
TNF-α	ACGTGGAACTGGCAGAAGAG	GGTCTGGGCCATAGAACTGA
IL-1β	TACAGGCTCCGAGATGAACA	AGGCCACAGGTATTTTGTCG
IL-6	AACGATGATGCACTTGCAGA	CTCTGAAGGACTCTGGCTTTG
iNOS	GGCTGTGCTCCATAGTTTCC	CAACATCTCCTGGTGGAACA
TBP	GCACAGGAGCCAAGAGTGAA	TCACAGCTCCCCACCATGTT

### Immunoprecipitation and immunoblotting analysis

Cells were lysed with lysis buffer (200 mM Tris-HCl (pH 7.5), 1.5 M NaCl, 10 mM EDTA, 25 mM sodium pyrophosphate, 10 mM glycerolphosphate, 10 mM sodium orythovanadate, 50 mM NaF, 1 mM PMSF, in combination with protein inhibitor cocktail). Total cell lysates were incubated with the indicated antibodies in combination with protein A/G beads (Santa cruz) for 3h. The complexes were washed five times with cell lysis buffer. The samples were subjected to SDS-PAGE and transferred onto nitrocellulose membranes. Blots were probed with the specific primary antibodies. After extensive washing, blots were then incubated with horseradish peroxidaseconjugated secondary antibody (Pierce) and visualized by chemiluminescence.

### Native PAGE

A 10% Native gel was pre-run with 25 mM Tris and 192 mM glycine for 30 min at 40 mA. Samples in the native sample buffer (62.5 mM Tris-HCl, pH 6.8, 20% glycerol, 0.02% Bromophenol Blue) were applied to the gel and electrophoresed at 25 mA at 4 °C until the bromophenol blue dye front reach the bottom of the gel. This was followed by Western blotting with the indicated antibodies.

### *In Vitro* Ubiquitination Assay

HEK 293T cells were transfected with Myc-TRAF6 and incubated overnight. Then TRAF6 was immunopurified with anti-TRAF6 and proteinA/G-Sepharose beads from HEK 293T cell lysates. Then the autoubiquitination assay was performed in a 50 μL reaction volume. Briefly, the immunoprecipitated TRAF6 was preincubated with various does of MED on ice for 30 min, and then 0.15 μg of recombinant E1-activating enzyme, 1 μg of Ubc13/Uev1a-conjugating complex, and 2.5 μg of ubiquitin were added. Four mM ATP was added to initiate the reaction, and the mixtures were incubated at 30 °C for 1 h with gentle agitation. Then the reaction was blocked by loading buffer and then the samples were subjected to immunoblotting with the indicated antibodies.

### LPS-induced endotoxin shock

Mice were i.p. pretreated with DMSO or MED (15 mg/kg) for 30 min and then i.p. injected with LPS (10 mg/kg). Body temperatures of mice were measured at 1, 4, 6, 10, and 24 h after LPS injection, respectively; meanwhile, the survival rates of mice were recorded at 12, 24, 36, 48, 60, and 72 h post-injection. For serum cytokine assay, mice were anesthetized and sacrificed at 8 h after LPS injection, and whole bloods of mice were collected and centrifuged to obtain serum. For cytokine mRNA assay, mice were pretreated with MED for 30 min, and then liver and peritoneal macrophages were collected for harvesting mRNA at 2 h after LPS injection.

### Statistical analysis

Data were collected from several independent experiments, with three replicates per experiment. All data were analyzed with two-tailed Student's *t* test in SPSS 11.0 and p*<*0.05 was considered statistically significant. Bars in the graph represent standard deviation (S.D.).

## Supporting Information

Figure S1 **MED suppresses DNA binding activity of NF-κB induced by LPS.**
(TIF)

Figure S2 **The effect of TRAF6 knockdown on activation of NF-κB and MAPK induced by LPS.**
(TIF)

Figure S3 **MED alone has no effect on body temperatures and cytokine levels in mice.**
(TIF)

Figure S4 **Effect of MED on the ubiquitination of IκBα in HEK 293T cells.**
(TIF)

Figure S5 **HPLC analysis for the purity of MED.**
(TIF)
