# Correction: Mycoepoxydiene Inhibits Lipopolysaccharide-Induced Inflammatory Responses through the Suppression of TRAF6 Polyubiquitination

**DOI:** 10.1371/annotation/9a2fb76a-b2c3-43b4-a0b1-e8ae773378b7

**Published:** 2012-11-01

**Authors:** Qiang Chen, Tenghui Chen, Wenjiao Li, Wei Zhang, Jingwei Zhu, Yang Li, Yaojian Huang, Yuemao Shen, Chundong Yu

There was an error in the title of the article, which omitted the word "Suppression." The correct title should read: Mycoepoxydiene Inhibits Lipopolysaccharide-Induced Inflammatory Responses through the Suppression of TRAF6 Polyubiquitination.

The correct citation is: Chen Q, Chen T, Li W, Zhang W, Zhu J, et al. (2012) Mycoepoxydiene Inhibits Lipopolysaccharide-Induced Inflammatory Responses through the Suppression of TRAF6 Polyubiquitination. PLoS ONE 7(9): e44890. doi:10.1371/journal.pone.0044890 

