# Supplementary figures and images for: Mycoepoxydiene Inhibits Lipopolysaccharide-Induced Inflammatory Responses through the of TRAF6 Polyubiquitination
Source: PLoS One. 2012 Sep 11;7(9):e44890. doi: 10.1371/journal.pone.0044890 (PMC3439433; doi:10.1371/journal.pone.0044890)

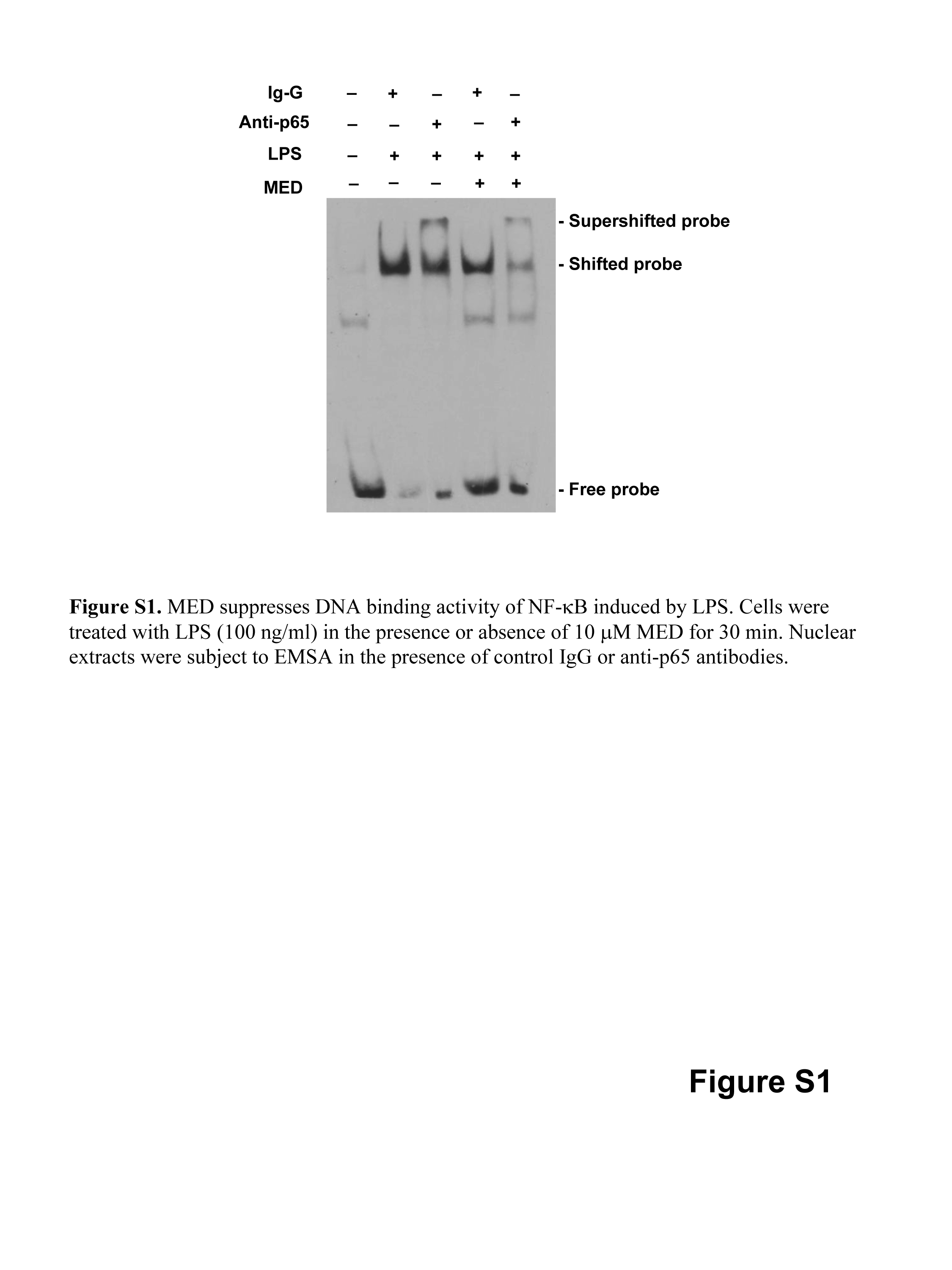

Supplement: Figure S1 — MED suppresses DNA binding activity of NF-κB induced by LPS. (TIF) [file pone.0044890.s001.tif]

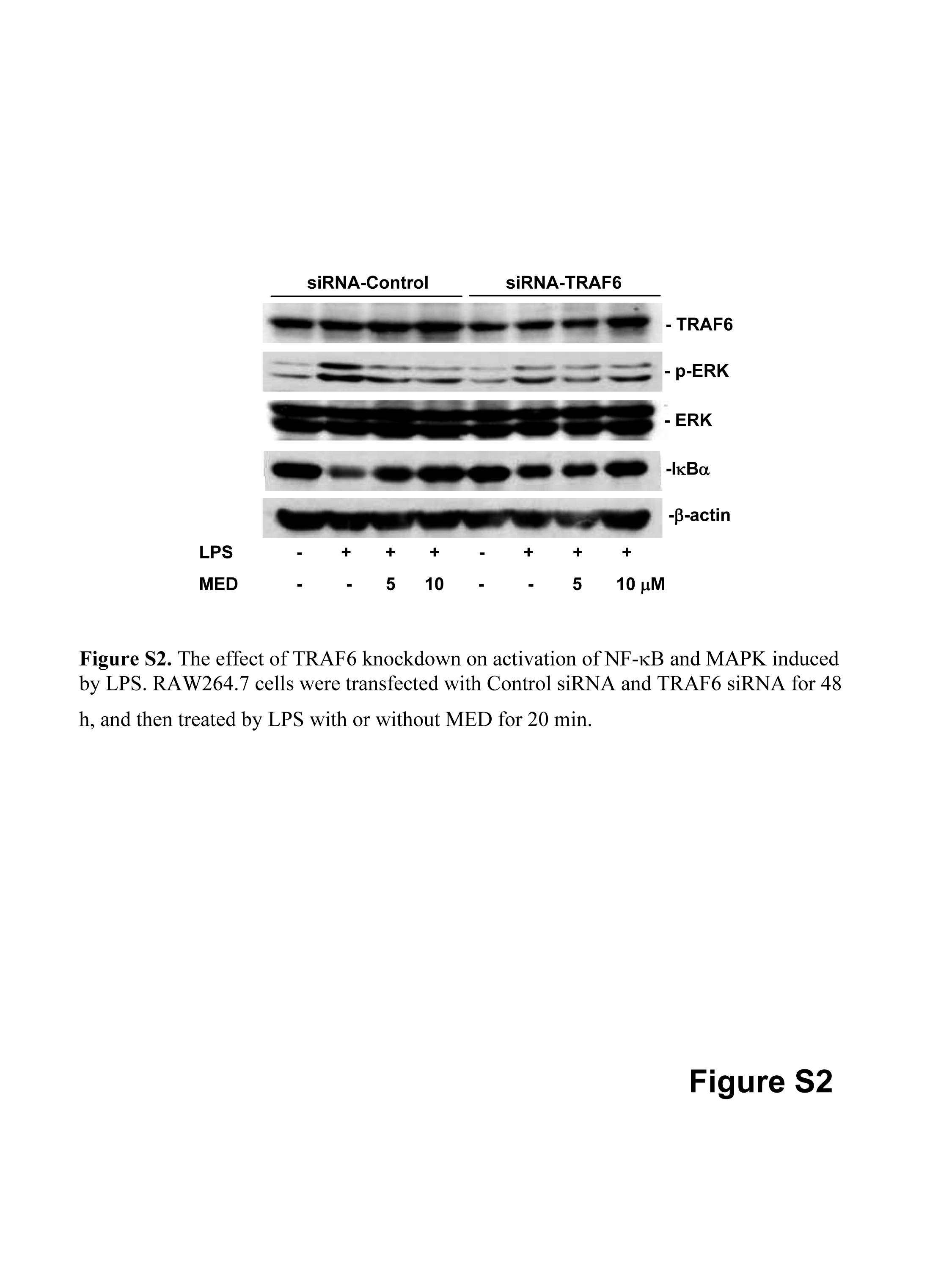

Supplement: Figure S2 — The effect of TRAF6 knockdown on activation of NF-κB and MAPK induced by LPS. (TIF) [file pone.0044890.s002.tif]

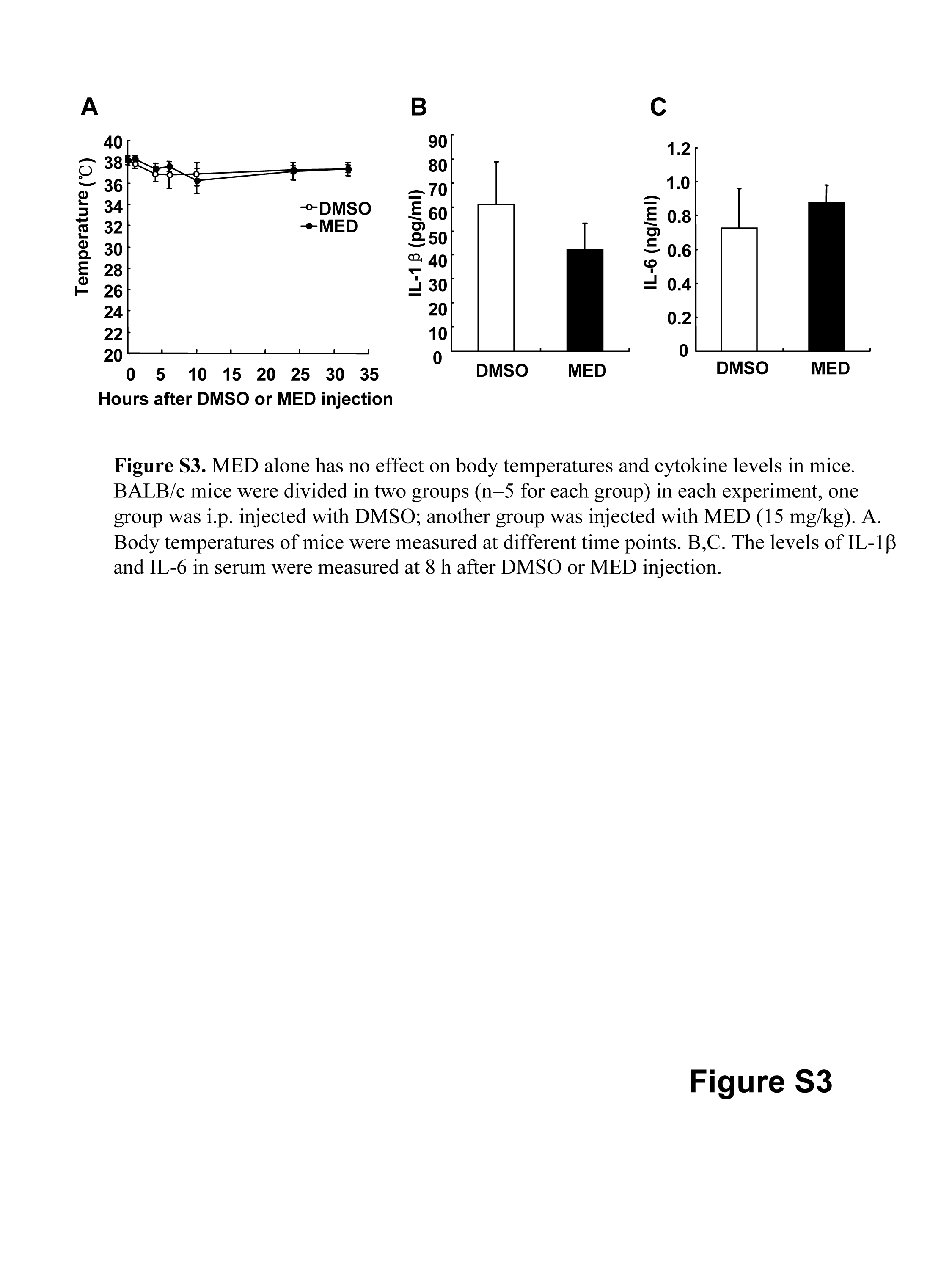

Supplement: Figure S3 — MED alone has no effect on body temperatures and cytokine levels in mice. (TIF) [file pone.0044890.s003.tif]

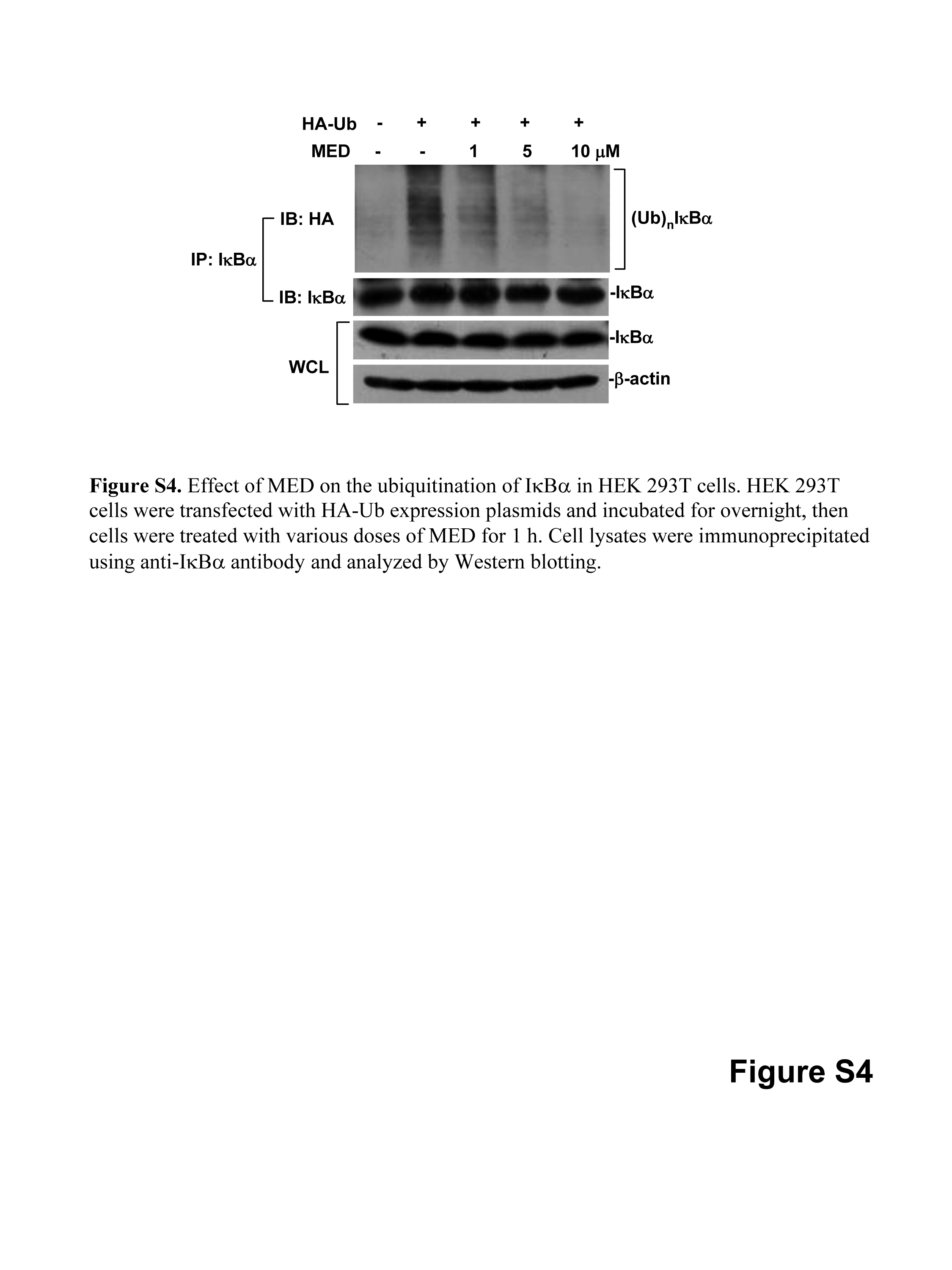

Supplement: Figure S4 — Effect of MED on the ubiquitination of IκBα in HEK 293T cells. (TIF) [file pone.0044890.s004.tif]

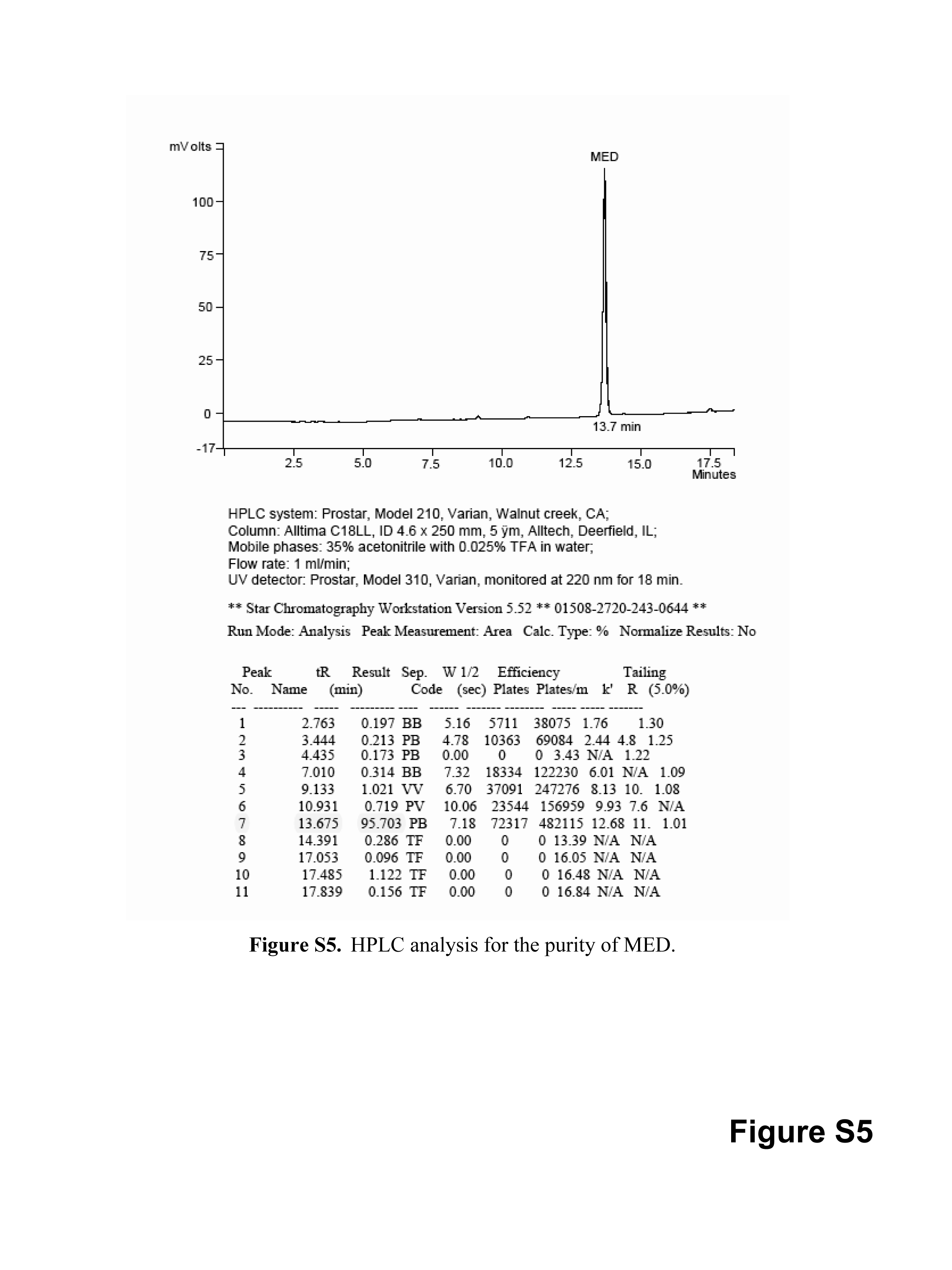

Supplement: Figure S5 — HPLC analysis for the purity of MED. (TIF) [file pone.0044890.s005.tif]
